# Automated treatment planning for whole breast irradiation with individualized tangential IMRT fields

**DOI:** 10.1002/acm2.14361

**Published:** 2024-04-20

**Authors:** Giulianne Rivelli Rodrigues Zaratim, Ricardo Gomes dos Reis, Marcos Antônio dos Santos, Nathalya Ala Yagi, Luis Felipe Oliveira e Silva

**Affiliations:** ^1^ Department of Radiation Oncology CONFIAR Radiotherapy Goiânia Goiás Brazil; ^2^ Department of Radiation Oncology University Hospital of Brasilia Brasilia Federal District Brazil

**Keywords:** artificial intelligence, automated planning, breast cancer, radiation therapy

## Abstract

**Purposes:**

This study aimed to develop and validate algorithms for automating intensity modulated radiation therapy (IMRT) planning in breast cancer patients, with a focus on patient anatomical characteristics.

**Material and Methods:**

We retrospectively selected 400 breast cancer patients without lymph node involvement for automated treatment planning. Automation was achieved using the Eclipse Scripting Application Programming Interface (ESAPI) integrated into the Eclipse Treatment Planning System. We employed three beam insertion geometries and three optimization strategies, resulting in 3600 plans, each delivering a 40.05 Gy dose in 15 fractions. Gantry angles in the tangent fields were selected based on a criterion involving the minimum intersection area between the Planning Target Volume (PTV) and the ipsilateral lung in the Beam's Eye View projection. ESAPI was also used to gather patient anatomical data, serving as input for Random Forest models to select the optimal plan. The Random Forest classification considered both beam insertion geometry and optimization strategy. Dosimetric data were evaluated in accordance with the Radiation Therapy Oncology Group (RTOG) 1005 protocol.

**Results:**

Overall, all approaches generated high‐quality plans, with approximately 94% meeting the acceptable dose criteria for organs at risk and/or target coverage as defined by RTOG guidelines. Average automated plan generation time ranged from 6 min and 37 s to 9 min and 22 s, with the mean time increasing with additional fields. The Random Forest approach did not successfully enable automatic planning strategy selection. Instead, our automated planning system allows users to choose from the tested geometry and strategy options.

**Conclusions:**

Although our attempt to correlate patient anatomical features with planning strategy using machine learning tools was unsuccessful, the resulting dosimetric outcomes proved satisfactory. Our algorithm consistently produced high‐quality plans, offering significant time and efficiency advantages.

## 1 INTRODUCTION

The development of systems for automating radiation treatment planning has witnessed substantial growth in recent years.^1^, ^2^, ^3^, ^4^, ^5^, ^6^, ^7^, ^8^ These systems possess the capacity to alleviate the planner's workload, foster planning standardization, and reduce dependence on the planner's experience. Furthermore, automation tools have shown promise in enhancing patient access to advanced treatments like Intensity Modulated Radiation Therapy (IMRT) while concurrently reducing costs and resource utilization, as demonstrated by several studies.^3^, ^9^, ^10^, ^11^

Application Programming Interfaces (APIs) serve as valuable tools, enabling seamless integration with the Treatment Planning System (TPS), thus facilitating automation across various facets of radiotherapy, including file transmission, plan quality assessment, and even treatment planning, as highlighted by Kim et al.^12^ Furthermore, as elucidated by Cardan, these capabilities represent potent extensions that greatly expedite research, particularly in scenarios involving repetitive tasks within extensive patient cohorts.^13^ Additionally, K. Chen et al.’s example illustrates how these tools offer the opportunity to establish a standardized automation workflow without depending on prior databases contingent upon the planner's expertise, a common limitation encountered with machine learning methods.^9^

In comparison to other cancer sites, treatment planning for breast irradiation is comparatively straightforward, yet it accounts for a significant portion of the workload in radiotherapy departments, rendering it an ideal candidate for automation.^1^ However, the automation of breast planning has some particularities that can be difficult to overcome, such as the automatic choice of gantry angles for treatment with tangent fields.^5^, ^8^ This difficulty arises from the anatomical variation between patients, which makes it difficult to adopt a single arrangement of fields to automate treatment planning.^14^

Some authors associate the anatomical characteristics of patients with dose variations in organs at risk, such as the heart, ipsilateral lung, and contralateral breast.^15^, ^16^, ^17^, ^18^ Kundrát et al. concluded in their study that the relationship between dose differences in the ipsilateral lung can be explained by some anatomical features and that this can be used to identify simple and complicated anatomies when developing individualized treatment plans.^18^ Thus, the use of anatomical parameters in the automation of treatment planning may be relevant to obtain more individualized results even when automation is used.

Our goal was to develop algorithms for the automation of Intensity Modulated Radiation Therapy planning for breast cancer patients using the ESAPI tool, considering the patients’ anatomical characteristics. Herein we describe the techniques we used for automation and the dosimetric results of a planning study.

## 2 MATERIAL AND METHODS

We employed random selection to choose 400 female breast cancer patients undergoing breast‐conserving surgery without lymph node involvement for inclusion in our study. Patients with pacemakers were excluded. All research activities utilized anonymized patient data. The dataset consisted of 200 patients with left‐sided breast cancer and 200 with right‐sided breast cancer, all of whom underwent whole breast irradiation. Computed tomography (CT) scans were acquired in the free‐breathing state, with patients in the head‐first supine position on a breast board, and their arms positioned overhead. Our treatment protocol adhered to RTOG 1005 guidelines.^19^ The Eclipse treatment planning system (Varian Medical Systems, Palo Alto, California), version 16 was used, along with the Halcyon 3.0 treatment engine, also from Varian Medical Systems. Dose calculations were performed using the Analytical Anisotropic Algorithm (AAA) version 16.1, leaf motion calculations with the Smart Leaf Motion Calculator (SMLC) version 16.1, and optimization with the Photon Optimizer (PO) version 16.1. The computing hardware employed in this study utilized dual Intel(R) Xeon(R) Silver 4110 CPUs, each operating at a clock speed of 2.10 GHz.

### 2.1 Patient segmentation

Manual contouring was employed to delineate the Clinical Target Volumes (CTV), as proposed by the RTOG consensus,^20^ and a uniform margin of 5 mm was applied to establish the Planning Target Volumes (PTV). As we utilized retrospective data, all target volumes were revised by two radiation oncologists, each with over five years of experience. The organ‐at‐risk segmentation was assisted by the MVision AI Segmentation Service from MVision AI Oy (Helsinki, FI), followed by a review and final adjustments by the radiation oncologists.

### 2.2 Automation of planning

**FIGURE 1:**
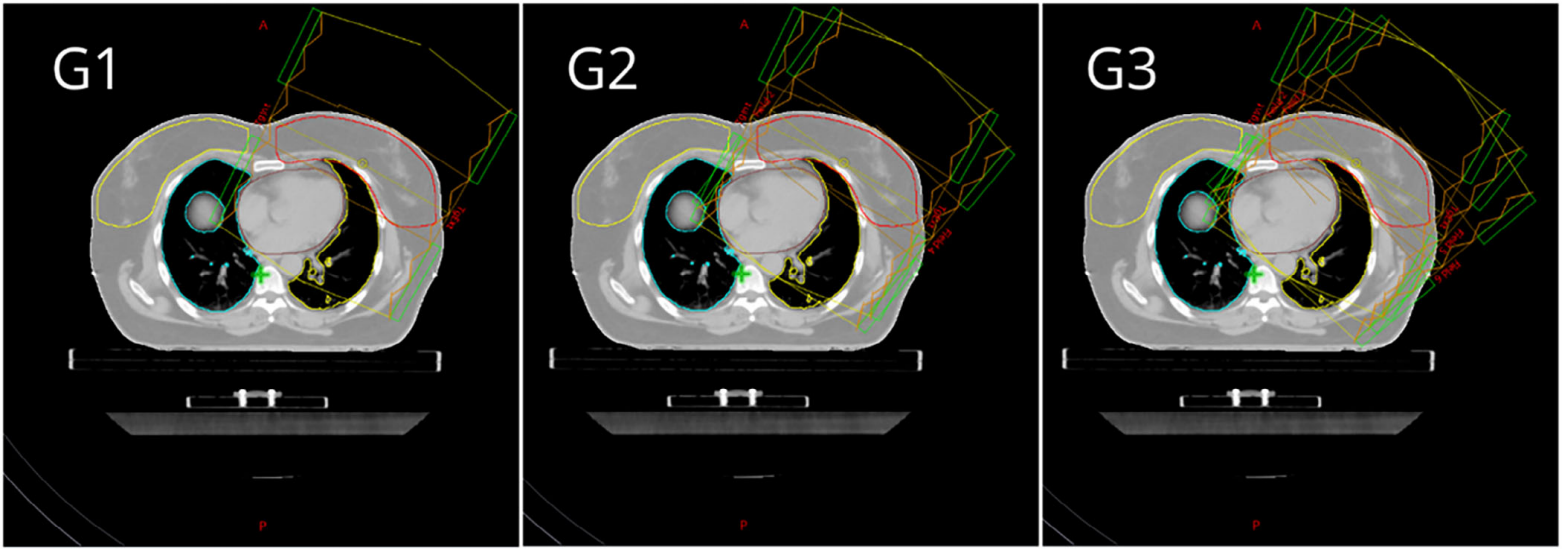
Representation of the beam insertion geometries.

We implemented three optimization strategies in conjunction with three field insertion geometries. Firstly, we developed an algorithm to automatically determine radiation field angles. This involved calculating the intersection area between the planning target volume (PTV) and the ipsilateral lung for various angles in the Beam's Eye View projection.^21^ For medial tangents, angles were explored between 295−330° for the left breast and 25−70° for the right breast. Lateral tangents were considered between 110−150° for the left breast and 200−245° for the right breast. The algorithm selected tangent field angles that resulted in the smallest calculated intersection area. Three different field insertion geometries were tested: G1 – tangent fields, G2 – tangent fields with a 10° angled field (clockwise for left, counterclockwise for right), and G3 – tangent fields with two 10° angled fields for each (clockwise for left, counterclockwise for right). The three geometries are depicted in Figure 1.

To achieve target coverage and adhere to dose constraints, we tested three different optimization strategies (OpS) that differed in initial objective weights. All strategies were derived from the dose tolerances specified in Radiation Therapy Oncology Group (RTOG) 1005 guidelines. For each field insertion geometry, we evaluated three optimization strategies, resulting in nine planning strategies. We use the notation GiOpSj, where ‘i’ represents the geometry index, ranging from 1 to 3, and ‘j’ represents the optimization strategy index, also ranging from 1 to 3, with strategy 1 being the least restrictive and 3 being the most restrictive.

In response to disparities in dose predictions between the optimization algorithm (PO) and the final dose calculation algorithm (AAA) (convergence error^22^), the algorithm employs an iterative process following the initial optimization and AAA dose calculation. This iterative cycle retains the original optimization objectives. Subsequently, the algorithm assesses compliance with the dose constraints outlined in the RTOG 1005 guidelines. If any objective is not met, a subsequent optimization is executed, involving adjustments to priority settings (objective weights). A subsequent evaluation follows, once again after AAA dose calculation. These adjustments are guided by the discrepancies observed between the dose constraints defined in RTOG 1005 and the doses computed by the AAA algorithm. This entire procedure is repeated once more, albeit with a focus on refining the doses specified within the optimization objectives. In the optimization process, we abstained from utilizing the TPS Intermediate Dose tool, and the calculation grid was set at 2.5 mm.

### 2.3 Automated plan evaluation

Considering the difference in RTOG 1005 heart dose constraints for left and right breast treatment, which necessitates distinct planning strategies for each, evaluations were conducted separately for each side. We assessed compliance with treatment protocol constraints by calculating medians, first quartiles, and third quartiles for all planning strategies. Statistical analysis was performed using Wilcoxon signed‐rank tests with the SciPy library in the Python programming language.

### 2.4 Selection of best planning strategy

To tailor our approach to individual patient anatomical characteristics, we developed a model to select the optimal plan from these 9 possibilities for each patient. Our goal was to streamline treatment planning by preselecting the optimization strategy and beam setup based on patient‐specific anatomical parameters using machine learning tools. We explored the relationship between dosimetric data and patient anatomy using Random Forest (RF) models.

For the anatomical characterization of the patients in the study, we used ESAPI to collect nine anatomic features. The parameters used for this description were: (1) the breast width (BW) of the treated breast, defined by the size of the straight‐line segment that passes through the isocenter and connects the field entry points of the medial and lateral tangents; (2) the ratio of the thorax anterior–posterior axis to latero‐lateral axis, thorax ratio (TR), measured in the ipsilateral lung midplane; (3) the mean intersection area (MIA), defined by the mean value of the intersection area between the PTV and the ipsilateral lung of the medial and lateral tangents in the beam's eye view projection; (4) the PTV volume (PV); (5) the ipsilateral lung volume (ILV); (6) the heart volume (HV); (7) the minimum distance between the PTV and the heart, PTV‐heart minimum distance (PHMD), measured in the heart midplane; (8) the PTV‐ipsilateral lung center‐point distance (PILCPD), obtained as the distance between the center of mass of each structure and (9) the contralateral breast PTV‐contralateral breast minimum distance (PCBMD), measured in the contralateral breast midplane.

To acquire the datasets essential for constructing the Random Forest (RF) models, we initially developed a Python algorithm that enumerated treatment plans meeting either the ideal constraints for each patient or, in cases where such plans were unavailable, acceptable constraints. Subsequently, we employed a hierarchical approach guided by the following criteria to identify the optimal plan:

1. Minimization of the mean heart dose, constrained within a range.
2. For left‐sided breast cancers, minimization of the dose for 5% of the heart volume, while for right‐sided breast cancers, minimization of the maximum heart dose.
3. Reduction of the heart volume fraction receiving 8 Gy.
4. Maximization of PTV volume fraction receiving at least 38 Gy.
5. Reduction of the ipsilateral lung volume fraction receiving 16 Gy.
6. Reduction of the ipsilateral lung volume fraction receiving 8 Gy.
7. Reduction of the ipsilateral lung volume fraction receiving 4 Gy.

To create training and testing datasets, we applied a 70/30 ratio division.

For each breast laterality, we developed two models, one for predicting the best geometry and another for predicting the best optimization strategy. Notably, we observed limited usage of G3 for right‐sided breast cancer patients, prompting a reevaluation without dosimetric data from this geometry.

We employed Exhaustive Feature Selection to identify the most effective feature subset for our final models. With this method all possible combinations of the mentioned anatomical features were tested. Parameters such as the number of estimators and maximum tree depth were varied across values ranging from 50, 100, 250, 500, and 1000 for the number of estimators, and from 1 to 15 for the maximum tree depth. Furthermore, in order to assess the issue of overfitting, cross‐validation was applied using K‐Fold with a number of splits equal to 3. The metric employed for evaluation was the mean precision. To avoid the overweighting of some features over others, we used Min‐Max scaling method, witch linearly transforms each feature within a specified range (0 to 1) by subtracting the minimum feature value and dividing by the range of feature values. Model evaluation included metrics such as average precision, accuracy, recall, and f1‐score. Additionally, a holdout method was employed, in which a separate test set, consisting of 1/4 of the data, was used for evaluation. This test set was not used during the training or cross‐validation.

### 2.5 Graphical user interface

**FIGURE 2:**
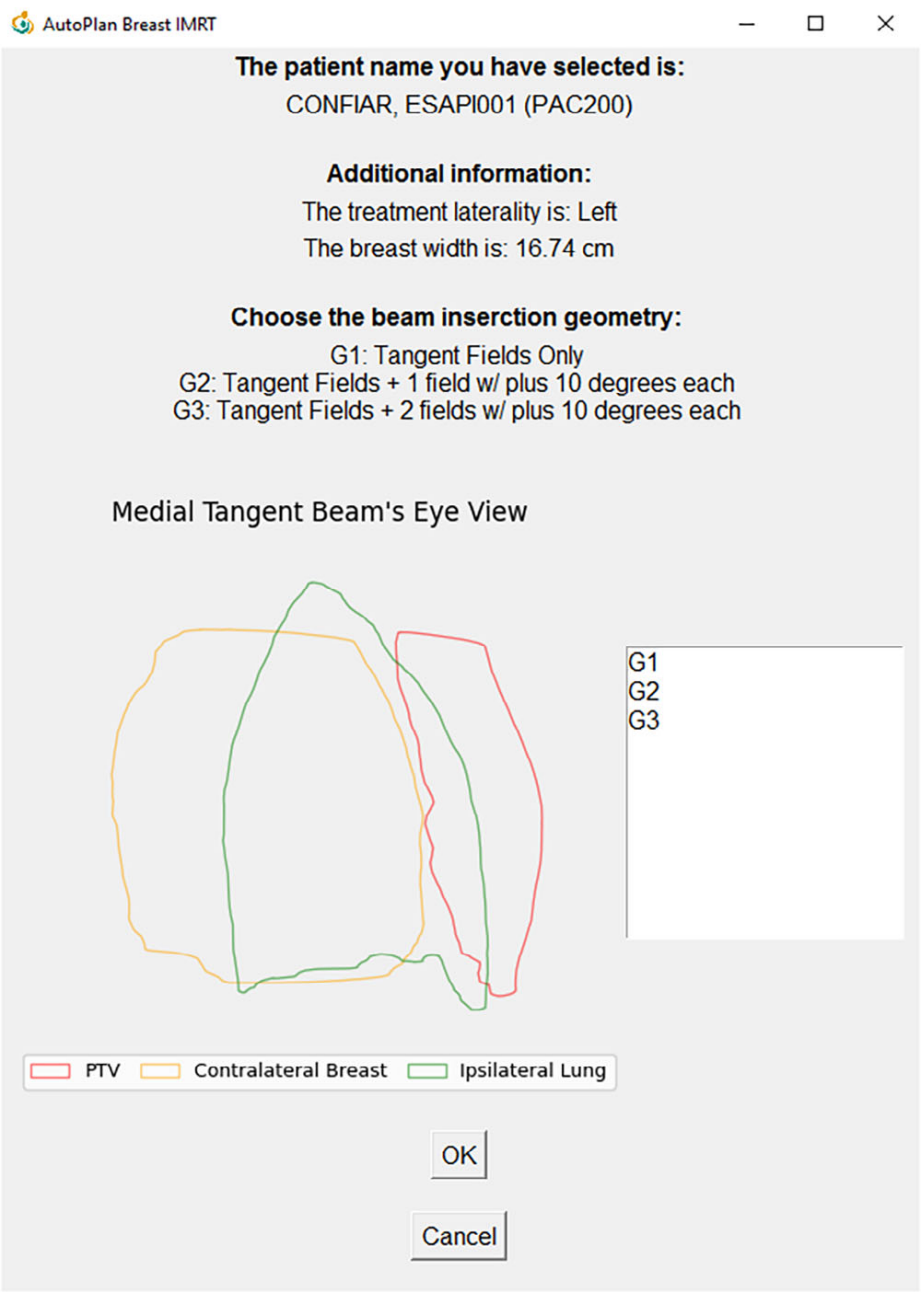
Graphical user interface.

To facilitate clinical use, we developed a Graphical User Interface using the Tkinter^23^ library in Python language. The communication between the Visual Studio and the Python environment was made using the Python.NET. The user can specify the patient ID, and the code returns the structure sets available so the user can associate the structures by its name, after the automatic detection of treatment laterality. The final window displays patient information, including the patient's name, laterality detected based on the structure set, breast width, and the available geometries (G1–G3). Additionally, it provides a medial tangent beam's eye view, allowing the user to select one or more geometries for the automatic plan, as illustrated in Figure 2. Moreover, the system allows for the selection of an arbitrary number of patients to be planned simultaneously.

## 3 RESULTS

### 3.1 Patient characteristics

The anatomical characteristics of the 400 patients in our data sample are presented in Table 1. The mean breast volume was 905 (±378) cc and 926 (±368) cc for right‐sided and left‐sided breast, respectively.

**TABLE 1:** Anatomical parameters for the 200 right‐sided patients and the 200 left‐sided patients presented as mean value (minimum–maximum value).

Laterality	BW (cm)	TR	MIA (cm^2^)	PV (cm^3^)	ILV (cm^3^)	HV (cm^3^)	PHMD (cm)	PILCPD (cm)	PCBMD (cm)
Right	18.01 (11.59–28.13)	1.63 (1.32–2.12)	2616.88 (508.68–4670.39)	1040.25 (331.50–2242.09)	1345.37 (887.86–2287.82)	579.91 (370.69–946.75)	2.13 (0.10–4.33)	8.06 (5.66–11.53)	2.74 (0.18–6.64)
Left	18.31 (11.90–29.66)	1.63 (1.30–2.02)	2415.61 (407.01–5557.22)	1053.83 (309.41–2504.90)	1082.42 (583.74–1977.47)	584.08 (328.50–1100.63)	0.71 (0.00–2.64)	8.34 (5.39–11.58)	2.88 (0.14–10.57)

Abbreviations: BW, breast width; TR, thorax ratio; MIA, mean intersection area; PV, PTV volume; ILV, ipsilateral lung volume; HV, heart volume; PHMD, PTV‐heart minimum distance; PILCPD, PTV‐ipsilateral lung center‐point distance; PCBMD, PTV‐contralateral breast minimum distance.

### 3.2 Automated plans

**FIGURE 3:**
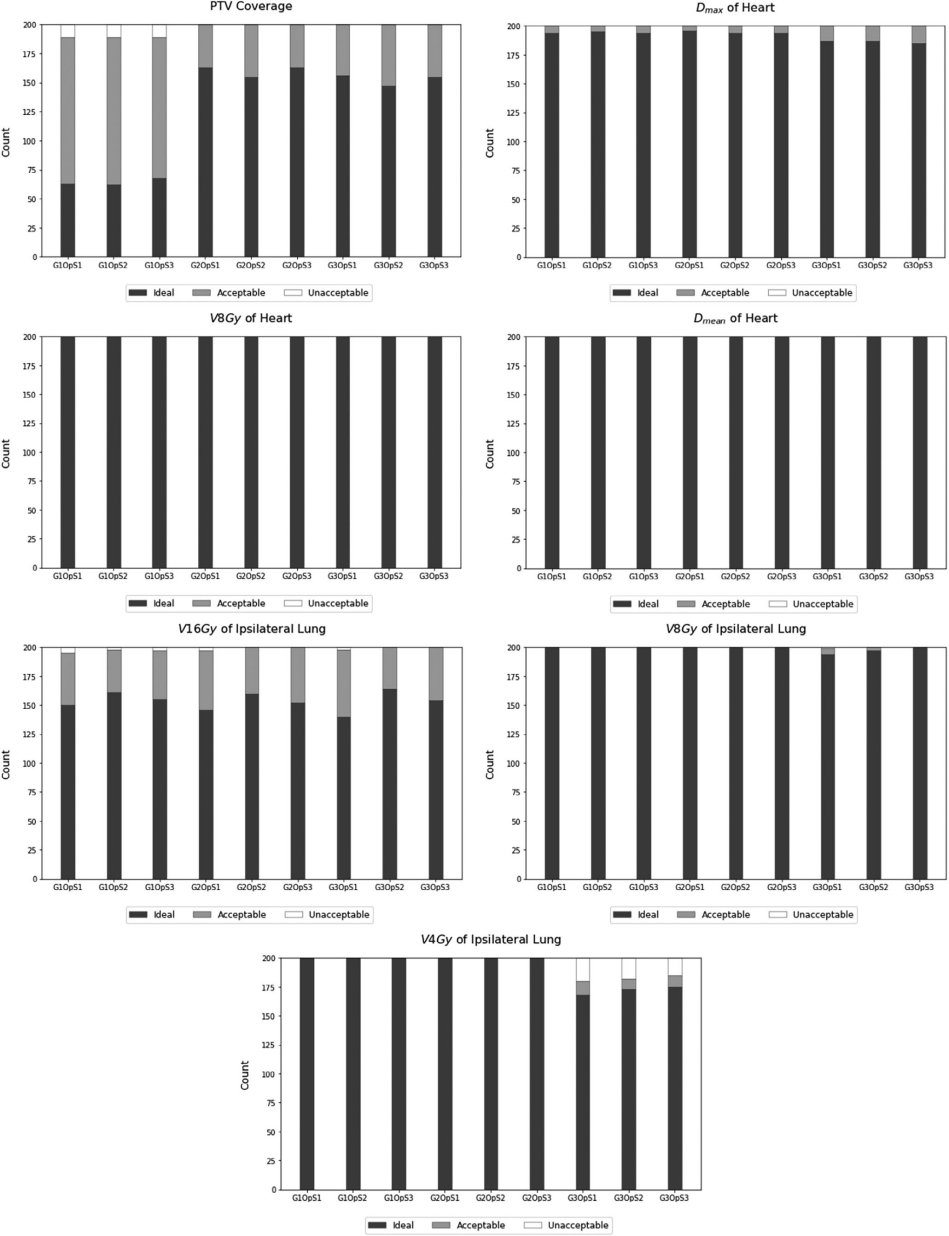
Number of automated treatment plans for right‐sided patients classified as ideal, acceptable, and unacceptable for each dosimetric parameter.

**FIGURE 4:**
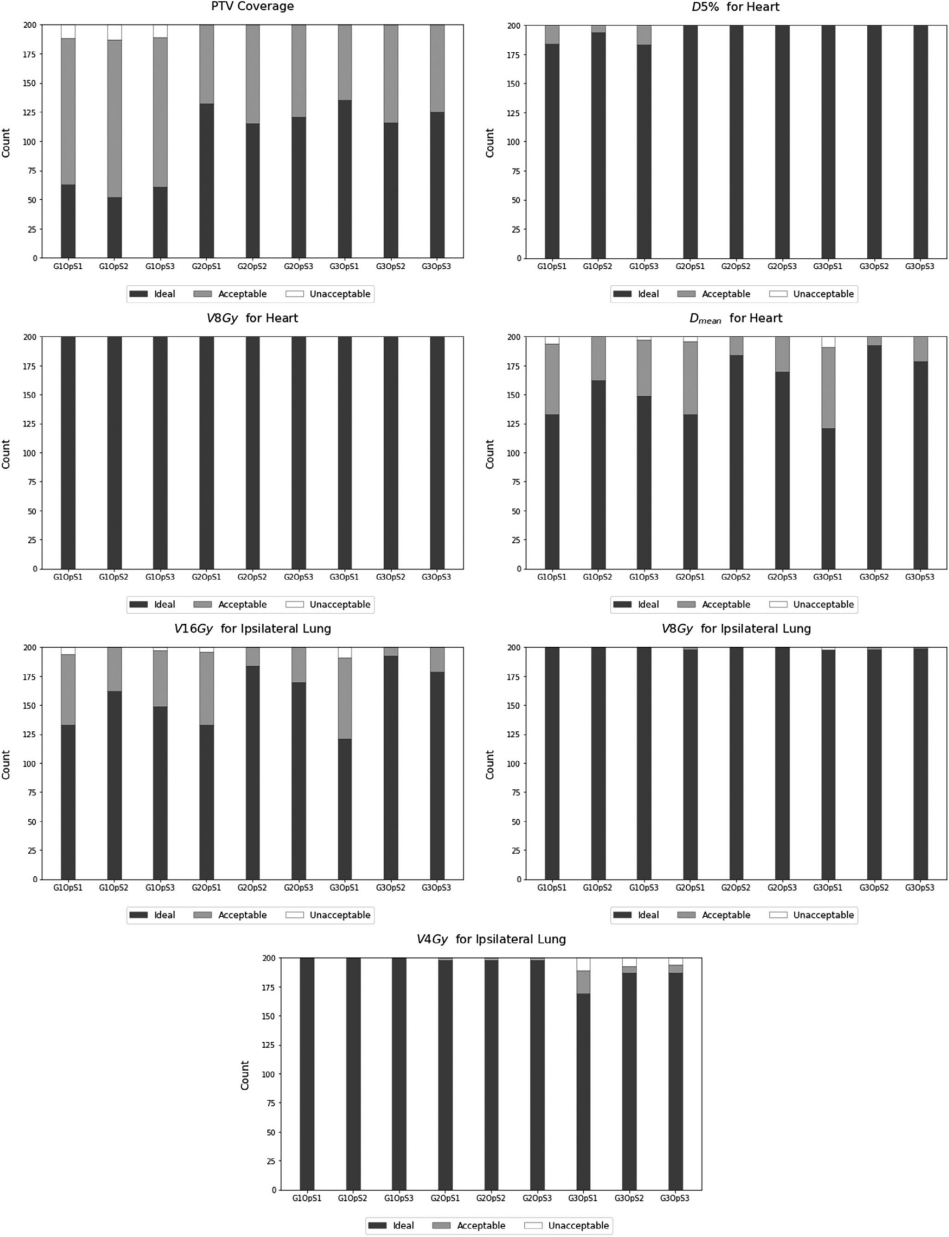
Number of automated treatment plans for left‐sided patients classified as ideal, acceptable, and unacceptable for each dosimetric parameter.

We have generated 3600 automatic plans to evaluate nine planning approaches. Overall, all approaches generated high‐quality plans, with approximately 94% meeting the acceptable dose criteria for organs at risk and/or target coverage as defined by RTOG 1005 guidelines. Figures 3 and 4 presents the number of plans with ideal, acceptable, and unacceptable dosimetric parameters, in accordance with RTOG standards.

Figure 3, concerning the approval count for right breast patients, indicates that Geometry G1, comprising solely tangent fields, encountered difficulties in achieving the acceptable coverage set by the protocol. For heart dose tolerances, all planning strategies successfully met the protocol's acceptable constraints. Regarding the ipsilateral lung, the three strategies incorporating G1 and the other two strategies employing OpS1 failed to meet the V16Gy for the ipsilateral lung. For V8Gy, the G1OpS3 strategy did not meet the acceptable constraint for one patient, and for V4Gy, none of the strategies with G3, consisting of tangent fields plus two auxiliary fields angled at every 10° per tangent, managed to ensure that all patients adhered to the dose tolerance. It is worth noting that, among right‐sided patients, both the G2OpS2 and G2OpS3 strategies displayed a greater number of plans meeting the dose constraints. Remarkably, none of the 200 patients exhibited any dosimetric parameter classified as unacceptable.

In Figure 4, which presents the plan approval count for left breast patients, we observed the same coverage difficulty for strategies generated by the field insertion geometry composed solely of tangent fields. Regarding the heart, challenges were encountered only with the average heart dose tolerance. Besides the three planning strategies in which optimization strategies OpS1 were used and failed to meet the constraint, the G1OpS3 strategy also fell short. For the V16Gy of the ipsilateral lung, none of the strategies managed to achieve the acceptable constraint for all patients; this occurred because, in one of the patients, none of the nine planning strategies could adhere to this dose tolerance. However, for two planning strategies, G2OpS2 and G3Ops2, the difficulty was limited only to this patient. For V8Gy, the only strategy that did not achieve the dose tolerance for all patients was G3Ops1. As for V4Gy, all strategies composed of six fields, G3, had difficulty meeting the acceptable constraint for all patients. Amongst left‐sided patients, it is evident that the G2OpS2 strategy yielded a higher number of plans that successfully adhered to the dose constraints. Nevertheless, we underscore that only one patient among the cohort posed a challenge, as none of the nine planning strategies could meet the V16Gy constraint for the ipsilateral lung.

The medians and interquartile ranges for the dosimetric parameters are presented in Tables 2 and 3. For both lateralities, it is noteworthy that the median values obtained for the planning strategies concerning the organs at risk are lower than what the protocol identifies as ideal. However, it is observable that the median values of the ideal coverages (V95) exceed the value established by the protocol for all strategies that include additional fields to the tangent fields; strategies with geometry consisting solely of tangent fields showed satisfactory medians only for acceptable coverage (V90). Notably, the medians for the maximum dose to the contralateral breast exceeded the acceptable value for all planning strategies. Upon applying the previously outlined hierarchical criteria to identify the optimal treatment plans within our cohort, the most favorable results were attained with Geometry 2 and Strategy 2 for right‐sided and for left‐sided breast cases.

**TABLE 2:** Median values and interquartile ranges of dosimetric parameters for our cohort of 200 right‐sided patients.

		Dose constraints									
		PTV			Heart			Ipsilateral Lung			Contralateral breast
Strategy		V95 (%)	V90 (%)	*D*_max_(%)	*D*_max_ (cGy)	V8Gy (%)	*D*_med_ (cGy)	V16Gy (%)	V8Gy (%)	V4Gy (%)	*D*_max_ (cGy)
G1OpS1	Median (IQR)	93.23 (4.16)	96.07 (3.70)	105.85 (0.73)	415.04 (113.40)	0.00 (0.00)	109.48 (19.98)	12.42 (4.57)	17.32 (4.86)	30.10 (7.37)	2036.97 (3456.89)
G1OpS2	Median (IQR)	93.20 (3.71)	96.09 (3.71)	105.96 (0.78)	406.14 (104.73)	0.00 (0.00)	108.93 (20.06)	12.11 (4.12)	16.86 (4.55)	29.58 (7.34)	1108.66 (2392.20)
G1OpS3	Median (IQR)	93.42 (4.44)	96.21 (3.71)	106.14 (0.77)	411.58 (107.57)	0.00 (0.00)	109.41 (20.84)	12.34 (4.25)	17.04 (4.72)	29.81 (7.32)	1241.04 (2654.57)
G2OpS1	Median (IQR)	97.30 (2.90)	99.27 (1.22)	105.71 (0.79)	411.85 (118.49)	0.00 (0.00)	106.79 (21.42)	13.25 (3.94)	20.25 (4.75)	33.40 (6.69)	1247.01 (2918.75)
G2OpS2	Median (IQR)	97.11 (3.17)	99.27 (1.26)	105.80 (1.00)	405.42 (117.23)	0.00 (0.00)	106.03 (21.85)	12.74 (3.37)	19.39 (4.21)	32.74 (6.40)	827.50 (1700.19)
G2OpS3	Median (IQR)	97.35 (2.89)	99.35 (1.24)	106.11 (0.92)	407.97 (117.86)	0.00 (0.00)	106.33 (21.91)	12.95 (3.77)	19.60 (4.24)	32.90 (6.37)	877.78 (1917.19)
G3OpS1	Median (IQR)	96.98 (3.00)	99.30 (1.10)	105.77 (0.92)	432.45 (310.62)	0.00 (0.00)	106.41 (24.13)	13.67 (24.13)	21.56 (6.74)	41.54 (13.29)	1024.58 (2563.28)
G3OpS2	Median (IQR)	96.92 (3.31)	99.28 (1.15)	105.82 (0.93)	423.86 (304.77)	0.00 (0.00)	105.40 (23.94)	12.93 (3.02)	20.18 (5.38)	40.09 (12.27)	761.61 (1453.36)
G3OpS3	Median (IQR)	97.17 (3.11)	99.30 (1.28)	105.97 (0.94)	421.71 (284.61)	0.00 (0.00)	105.57 (24.26)	13.13 (3.30)	20.16 (4.93)	39.25 (11.81)	824.45 (1718.40)

**TABLE 3:** Median values and interquartile ranges of dosimetric parameters for our cohort of 200 left‐sided patients.

		Dose constraints									
		PTV			Heart			Ipsilateral lung			Contralateral breast
Strategy		V95 (%)	V90 (%)	*D*_max_(%)	D5% (cGy)	V8Gy (%)	*D*_med_ (cGy)	V16Gy (%)	V8Gy (%)	V4Gy (%)	*D*_max_ (cGy)
G1OpS1	**Median (IQR)**	92.75 (6.55)	95.50 (4.06)	106.20 (1.20)	823.37 (825.47)	5.09 (4.08)	294.19 (82.81)	11.60 (5.16)	16.18 (5.80)	27.06 (8.61)	1249.16 (3320.23)
G1OpS2	**Median (IQR)**	92.58 (7.11)	95.52 (3.97)	106.34 (1.18)	666.79 (572.19)	4.49 (2.63)	282.98 (59.66)	11.14 (4.97)	15.55 (5.36)	26.64 (8.01)	757.54 (2224.52)
G1OpS3	**Median (IQR)**	92.96 (6.68)	95.61 (3.88)	106.52 (1.19)	704.53 (707.63)	4.66 (3.24)	288.12 (71.86)	11.34 (5.13)	15.72 (5.60)	26.84 (7.99)	792.64 (2381.97)
G2OpS1	**Median (IQR)**	96.19 (4.31)	99.04 (1.62)	106.08 (1.12)	1062.39 (487.95)	6.83 (4.08)	294.71 (65.09)	12.12 (4.35)	18.75 (5.02)	31.58 (7.95)	667.25 (2697.09)
G2OpS2	**Median (IQR)**	95.74 (4.86)	98.87 (1.74)	106.26 (1.27)	782.89 (328.15)	4.90 (1.80)	270.98 (31.62)	11.83 (3.80)	17.89 (4.56)	30.18 (7.49)	510.97 (1538.56)
G2OpS3	**Median (IQR)**	96.08 (4.70)	98.90 (1.85)	106.39 (1.39)	826.15 (396.18)	5.12 (2.00)	276.21 (41.65)	11.96 (4.15)	18.28 (4.99)	30.31 (7.63)	521.63 (1707.61)
G3OpS1	**Median (IQR)**	96.21 (4.06)	98.97 (1.62)	106.28 (1.34)	1042.49 (314.79)	7.68 (4.50)	309.84 (57.53)	12.31 (3.77)	19.60 (5.63)	39.12 (11.92)	536.94 (2425.78)
G3OpS2	**Median (IQR)**	95.64 (4.49)	98.74 (1.81)	106.44 (1.55)	768.04 (242.66)	4.81 (1.51)	267.67 (24.64)	12.02 (3.35)	18.89 (4.90)	35.65 (11.93)	451.83 (1361.69)
G3OpS3	**Median (IQR)**	95.98 (4.23)	98.81 (1.91)	106.64 (1.58)	799.64 (300.20)	5.00 (1.69)	271.88 (32.35)	12.13 (3.62)	18.71 (4.86)	35.22 (11.75)	481.29 (1599.15)

In the Appendix, the Tables A1 and A2 highlight the planning approaches that displayed statistically significant differences in median values for each dosimetric parameter. Among the strategies that produced the most favorable outcomes for patients with right‐side laterality, specifically G2OpS2 and G2OpS3, it was noted that the OpS3 optimization strategy, despite applying stricter dose constraints, resulted in a statistically significant improvement only in heart V8Gy values. However, the maximum heart V8Gy value observed with the G2OpS2 strategy was 1.22%, whereas for the G2OpS3 strategy, the highest recorded value was 1.15%. When comparing G2OpS2 against other planning strategies, a statistical difference was observed in the dose coverage parameters, V95 and V90, in the maximum dose within the PTV, in the V8Gy tolerance for the heart, and in the V16Gy for the ipsilateral lung. Nonetheless, in the comparison of G3OpS2, no statistical difference in terms of coverage was observed relative to any other strategy; differences were noted in the maximum dose of the PTV, the V8Gy and the average dose in the heart, and the V16Gy in the ipsilateral lung. No statistical difference was found between the dosimetric parameters of this strategy and the G3OpS1 strategy.

For the G2OpS2 strategy in patients with left‐side laterality, it is noteworthy that no dosimetric parameter evaluated showed a statistical difference compared to three strategies: G1OpS1, G2OpS1, and G2OpS3. In comparison to other strategies, differences were observed in the coverage parameters and maximum dose of the PTV. For the heart, the difference was only in the V5% and V8Gy parameters, and for the ipsilateral lung, the difference was noted in V16Gy.

The mean Monitor Units ranged from 803 to 1277, exhibiting an upward trend corresponding to the increment in the number of beams. The average time for generating the automated plan ranged from 6 min and 37 s to 9 min and 22 s, with the mean value increasing as more fields were added.

### 3.3 Selection of best planning strategy

After the exhaustive feature selection process, we present the results of the models with the best F1 scores in Table 4. For both right and left‐sided breasts, the final models were unable to satisfactorily predict the optimal planning geometry and strategy.

**TABLE 4:** Performance evaluation of the final random forest models.

	Classification	Anatomical Features	Depth	Number of estimators	Group	Precision	Recall	F1‐score	Accuracy	Mean Precision K‐Fold
**Right‐sided patients’ models**	**Geometry**	PV, MIA, PILCPD, PCBMD	4	50	Train	0,925	0,914	0,912	0,914	0,602
					Test	0,863	0,822	0,808	0,822	0,608
					Validation	0,760	0,760	0,760	0,760	–
	**Strategy**	BW, PV, ILV, HV, PILCPD	5	500	Train	0,948	0,943	0,939	0,943	0,573
					Test	0,746	0,800	0,770	0,800	0,585
					Validation	0,760	0,660	0,608	0,660	–
**Left‐sided patients’ models**	**Geometry**	BW, PV, ILV, HV, PILCPD	5	100	Train	0,935	0,933	0,933	0,933	0,411
					Test	0,671	0,667	0,667	0,677	0,450
					Validation	0,392	0,420	0,395	0,420	–
	**Strategy**	BW, PCBMD	5	50	Train	0,886	0,867	0,840	0,867	0,716
					Test	0,902	0,889	0,861	0,889	0,675
					Validation	0,496	0,680	0,573	0,680	–

Abbreviations: BW, breast width; TR, thorax ratio; MIA, mean intersection area; PV, PTV volume; ILV, ipsilateral lung volume; HV, heart volume; PHMD, PTV‐heart minimum distance; PILCPD, PTV‐ipsilateral lung center‐point distance; PCBMD, PTV‐contralateral breast minimum distance.

Despite the metrics yielding favorable results overall, an assessment of the cross‐validation reveals a low mean accuracy in the models, indicating insufficient generalization. Additionally, the metrics obtained when applying the selected models to the validation group suggest that the models are not sufficiently robust for clinical routine application.

Given that the Random Forest approach did not yield successful results, the automatic selection of the planning strategy was not implemented. Instead, in the automatic planning system developed in this study, the users can choose one of the options of geometry and strategy tested in this work.

## 4 DISCUSSION

The pursuit of developing automated planning systems has gained momentum in recent years.^1^, ^2^, ^7^, ^24^ This trend not only alleviates the workload of physicists and dosimetrists but also contributes to standardizing the planning process, reducing reliance on individual planner expertise. Additionally, automation tools offer the potential to enhance patient access to high‐quality IMRT treatments without necessitating an increase in treatment costs or the planners’ workload.^3^

Our study's analysis of diverse planning strategies for radiation therapy in patients with right‐sided and left‐sided breast cancer has yielded some observations. Specifically, for left‐sided patients, we encountered a significant challenge with only one patient for whom none of the nine planning strategies met the V16Gy constraint for the ipsilateral lung. In patients with right‐sided breast cancer, the implementation of the G2OpS2 and G2OpS3 strategies, each adding an extra radiation field per tangent, demonstrated that, across 200 patients, all dosimetric parameters were within acceptable limits. This observation suggests that the addition of two extra radiation fields may be unnecessary; instead, incorporating just one additional field for each tangent could suffice to achieve the desired outcomes in this particular clinical context.

The G2 geometry, by offering additional optimization flexibility beyond standard tangent fields, enabled the development of plans that adhere more closely to the guidelines set forth by the RTOG. Furthermore, the OpS3 optimization strategy implemented stricter dose constraints, resulting in a statistically significant improvement only in heart V8Gy values. However, this improvement did not achieve clinical significance in reducing the dose to this organ at risk compared to the OpS2 strategy, as the maximum heart V8Gy observed with this strategy was only 1.22%. Although a marginally higher number of plans met the ideal criteria with OpS3, this did not translate into a clinically significant difference in the median values of the evaluated dosimetric parameters.

The literature discusses several automation tools utilizing various Treatment Planning Systems.^1^, ^2^, ^3^, ^4^, ^5^, ^6^, ^7^, ^8^, ^25^ While there have been reports on the automation of breast irradiation planning, not all of them incorporate the automatic insertion of tangential fields into the process.^6^, ^8^, ^25^ Moreover, a one‐size‐fits‐all approach, not personalized, often employed in these plans can lead to compromised target volume coverage, higher doses to organs at risk, or constraints on the inclusion of tangential fields.^11^, ^26^

Purdie et al. automated the planning process for 158 breast cancer patients undergoing whole‐breast IMRT within the Pinnacle Treatment Planning System.^11^ While the algorithm efficiently handled many planning aspects, it was not suitable for cases requiring contralateral breast field entry, a limitation to consider when clinical decisions prioritize sparing the heart or ipsilateral lung. Therefore, the development of an automation tool that offers some degree of plan individualization without significantly increasing the workload could lead to improved automated plan quality.^27^ In our approach, besides tailoring plans to each patient's anatomical characteristics, we introduce a user‐friendly graphical interface for beam visualization and access to relevant information, enabling the user to select the optimal strategy.

Lin et al. employed ESAPI for planning automation in a study involving 99 left‐sided breast cancer patients undergoing post‐operative whole‐breast irradiation. The dosimetric results presented by the group were excellent. However, it is noteworthy that the study population was stratified based on breast size, with small breasts defined as <300 mL, large breasts as >600 mL, and medium‐sized breasts falling between these ranges.^1^ In our study, the mean volume of the Clinical Target Volume (CTV) was approximately 900 mL, and only a small number of patients exhibited volumes less than 300 mL (7/400). This discrepancy suggests anatomical differences between the study populations, which may contribute to the superior performance described in their study.

Some studies^28^, ^29^, ^30^, ^31^ harnessed the power of a knowledge‐based planning (KBP) methodology, a process that involves the training of a computational model on a database containing treatment plans from previously treated patients. The objective behind this approach is to enable the model to predict optimal treatment plans for new patients based on the collective wisdom encapsulated within the existing dataset. This strategy holds significant promise for streamlining the radiotherapy planning process, offering the potential to enhance efficiency and consistency.

However, despite the considerable advantages offered by KBP and machine learning in automating treatment planning, there are certain inherent limitations that merit attention. One notable limitation lies in the model's reliance on the quality of the plans used during its training phase. This means that the effectiveness of the automated planning process is intrinsically linked to the expertise and experience of the planners who originally generated the training plans. In cases where the training plans do not fully encapsulate the desired planning objectives, the model's predictive capabilities may be compromised.

Additionally, another challenge in employing machine learning models for plan automation is the requirement for a dedicated and well‐curated database for training. In situations where there is a change in the treatment protocol or the introduction of new planning objectives, the existing model may no longer suffice, necessitating the creation of a new database. This process can be resource‐intensive and time‐consuming, potentially impeding the adaptability of the planning system.

In contrast, the approach adopted in our study offers a distinct advantage. Our algorithm has been designed with flexibility in mind, allowing for easy adaptation to accommodate evolving planning goals or changes in treatment protocols. This adaptability ensures that our automated planning system can seamlessly align with the dynamic nature of clinical practice, offering a promising avenue for enhancing the efficiency and effectiveness of radiotherapy planning.

In our empirical observations, the utilization of Machine Learning models to establish correlations between anatomical patterns and their relevance to planning strategies did not yield promising outcomes or significantly contribute to the personalization of automated planning. The selection of parameters was guided by the insights gleaned from prior investigations,^16^, ^17^, ^18^ where associations between specific anatomical features and dosimetric data were noted.

Despite the initial promise suggested by these earlier studies, our findings underscore the complexity and multifaceted nature of the relationship between anatomical attributes and treatment planning. The Machine Learning models employed in our research were unable to discern nuanced patterns or reliably inform personalized planning strategies. This limitation may be attributed to the similarity in results among the strategies we developed.

This challenges the assumption that straightforward correlations between anatomy and planning can be established solely based on previously identified anatomical features. Our study suggests that a more comprehensive understanding of the interplay between various factors, including patient‐specific considerations, treatment objectives, and anatomical nuances, is necessary for the effective development of personalized and automated planning systems. Further research in this domain may yield insights that enhance the utility of Machine Learning in radiotherapy planning.

Observation reveals that the median maximum dose values in the contralateral breast exceeded the protocol recommendations (≤264 cGy) for both lateralities'. This outcome can be attributed to a higher prioritization of target coverage and the sparing of the heart and lungs in the context of the left breast, and target coverage and lung sparing in the case of the right breast. It is noteworthy that in our institution, plans generated manually usually present elevated maximum dose values in the contralateral breast, surpassing the recommended thresholds. This may reflect a common practice, grounded in the rationale of emphasizing target coverage while protecting the heart and lungs.

A study conducted by Liu et al., which involved a comparative analysis of automated and manual plans for IMRT planning, revealed that automated plans yielded lower maximum point dose values. Specifically, the automated plans resulted in 237 ± 1.02 cGy, whereas the non‐automated plans resulted in 423 ± 149 cGy. It is essential to clarify that the values reported in the study pertain to the dose received per 1.0 cubic centimeter (cc) and not the maximum dose. Additionally, the mean dose received by the heart in the study was 859 ± 237 cGy for the automated plans and 1043 ± 241 cGy for the manual plans.^32^ Notably, these values exceeded the recommended limits outlined by the RTOG1005 protocol (≤500 cGy). Furthermore, in the context of comparing each specific study arm that we adopted in our research to its counterpart within the same overarching study, the dose constraints for the heart and ipsilateral lung that we implemented were notably more stringent than those documented in the comparative study arm. For all dose tolerances specified in the protocol concerning these organs at risk, the mean values derived from all planning strategies in our investigation were found to be lower than the criteria established as ideal by Arm II of RTOG 1005, which was adopted in our study. In addition, the planning strategy that demonstrated superior performance for the left breast, within the same scope as the aforementioned work, revealed that the average values of the dose tolerances for the ipsilateral lung and heart were at least 20% and 16% lower, respectively, compared to the ideal thresholds specified by the protocol. This level of stringency is underscored by the fact that only one patient failed to meet the prescribed RTOG V16Gy constraint for the ipsilateral lung across all planning strategies.

Regarding the average planning time, the obtained value for all strategies was less than 10.0 min (ranging from 6.8 to 9.3 min). In the study, Liu et al. reported a planning time of 10.1 min, establishing that it is lesser than the time expended in non‐automated planning.^32^ Pudie et al. evaluating automated planning by heuristic optimization, found a planning time of 6.8 ± 1.2 min.^11^ In another study that evaluated automated planning in over 1600 patients, the time allocated for each planning session ranged from 5.0 to 6.0 min, further highlighting a reduction compared to manual planning time.^33^ Additionally, Guo et al. reported a time of 8.0 min for generating hybrid automated IMRT plans.^2^ Therefore, the planning time identified in our study aligns with the range of values reported in the scientific literature. It's essential to note that the planning time is influenced by hardware and the nature of the optimization and calculation algorithms used. Furthermore, it should be noted that the time required to generate an automatic plan could be further reduced with the implementation of technologies such as GPUs. However, such technology was not available in the computing stations where our research was conducted.

We have successfully created an automation tool that emulates the meticulousness of optimal manual treatment planning. Nevertheless, one noteworthy limitation of our algorithm lies in its inability to automate the insertion of skin flash, a task that users must execute manually after the algorithm's completion.

In addition to the previously mentioned standardization and reduction of workload for the planning teams, the automation of treatment plans can be highly significant for facilities utilizing Halcyon linear accelerators (Varian Medical Systems). This clinical accelerator model differs from the Series‐C models, primarily, by not having collimator jaws and a light field, and it only employs a flattening filter‐free (FFF) beam. These differences can pose challenges for its use in conventional techniques, potentially increasing planning and dose delivery time, depending on whether the beam flattening technique is used.^34^, ^35^

## 5 CONCLUSIONS

Our study introduces an automated planning algorithm designed for the optimized generation of whole‐breast radiotherapy treatment plans using ESAPI. This tool enables the creation of multiple plans customized to meet diverse clinical requirements through specially crafted algorithms. Additionally, its automation capabilities promote plan standardization, alleviate the planner's workload, and reduce the dependence on their expertise.

Although our attempt to correlate patient anatomical features with the planning strategy using machine learning tools was unsuccessful, the resulting dosimetric outcomes have proven to be satisfactory. Our algorithm consistently produced high‐quality plans, offering significant time and efficiency advantages.

In future studies, the automation of breast treatment planning will be extended to encompass breast with lymph node irradiation and breast treatment with simultaneous integrated boost (SIB).

## AUTHOR CONTRIBUTIONS

Giulianne Rivelli Rodrigues Zaratim: study conception, data collection, statistical analysis, manuscript writing, critical review, and final approval of the version to be published. Ricardo Gomes dos Reis: study conception, manuscript writing, critical review, and final approval of the version to be published. Marcos Antônio dos Santos: review of the structure outlines, guidance on the hierarchy used to generate the Random Forest model database, manuscript writing, critical review, and final approval of the version to be published. Nathalya Ala Yagi: review of the structure outlines, guidance on the hierarchy used to generate the Random Forest model database, manuscript writing, critical review, and final approval of the version to be published. Luis Felipe Oliveira e Silva: study conception, statistical analysis, manuscript writing, critical review, and final approval of the version to be published.

## CONFLICT OF INTEREST STATEMENT

The authors declare no conflicts of interest.

## Data Availability

The data that support the findings of this study are available on request from the corresponding author.

## APPENDIX A

**TABLE A1:** Automated plans exhibiting statistically significant differences in median values for each dosimetric parameter for right‐sided patients.

	Dose constraints									
	PTV			Heart			Ipsilateral Lung			Contralateral breast
Strategy	V95%	V90%	*D*_max_	*D*_max_	V8Gy	*D*_med_	V16Gy	V8Gy	V4Gy	*D*_max_
**G1OpS1**			G2OpS2, G3OpS1, G3OpS2		G1OpS3, G2OpS1, G2OpS2, G2OpS3		G3OpS2, G3OpS3			
**G1OpS2**			G2OpS3, G3OpS3	G2OpS1	G2OpS1, G2OpS2, G2OpS3		G2OpS2			G3OpS1
**G1OpS3**			G2OpS3, G3OpS3		G1OpS1, G2OpS1, G2OpS2, G2OpS3		G2OpS2, G2OpS3, G3OpS2			G2OpS1
**G2OpS1**		G2OpS2	G3OpS1	G1OpS2	G1OpS1, G1OpS2, G1OpS3	G3OpS2, G3OpS3		G3OpS3		G1OpS3
**G2OpS2**	G3OpS1, G3OpS3	G2OpS1	G1OpS1, G3OpS1, G3OpS2		G1OpS1, G1OpS2, G1OpS3, G2OpS3		G1OpS2, G1OpS3			
**G2OpS3**			G1OpS2, G1OpS3, G3OpS3		G1OpS1, G1OpS2, G1OpS3, G2OpS2	G3OpS2, G3OpS3	G1OpS3			
**G3OpS1**	G2OpS2	G3OpS3	G1OpS1, G2OpS1, G2OpS2							G1OpS2
**G3OpS2**		G3OpS3	G1OpS1, G2OpS2	G3OpS3		G2OpS1, G2OpS3	G1OpS1, G1OpS3			
**G3OpS3**	G2OpS2	G3OpS1, G3OpS2	G1OpS2, G1OpS3, G2OpS3	G3OpS2		G2OpS1, G2OpS3	G1OpS1	G2OpS1		

**TABLE A2:** Automated plans exhibiting statistically significant differences in median values for each dosimetric parameter for left‐sided patients.

	Dose constraints									
	PTV			Heart			Ipsilateral lung			Contralateral breast
Strategy	V95%	V90%	*D*_max_	D5%	V8Gy	*D*_med_	V16Gy	V8Gy	V4Gy	*D*_max_
**G1OpS1**			G2OpS1			G2OpS1	G2OpS1, G3OpS2, G3OpS3			
**G1OpS2**			G2OpS2, G3OpS1, G3OpS2	G2OpS2, G2OpS3, G3OpS2, G3OpS3	G2OpS2, G3OpS2					G3OpS1
**G1OpS3**			G2OpS2, G2OpS3, G3OpS1, G3OpS2, G3OpS3	G3OpS3	G2OpS3, G3OpS3		G2OpS2, G2OpS3			G3OpS1
**G2OpS1**	G3OpS1	G3OpS1	G1OpS1	G3OpS1		G1OpS1	G1OpS1	G3OpS2		
**G2OpS2**	G3OpS2	G3OpS2, G3OpS3	G1OpS2, G1OpS3, G3OpS1, G3OpS2	G1OpS2, G3OpS2	G1OpS2		G1OpS3			
**G2OpS3**	G3OpS1		G1OpS3, G3OpS2, G3OpS3	G1OpS2, G3OpS3	G1OpS3		G1OpS3, G3OpS2			
**G3OpS1**	G2OpS1, G2OpS3	G2OpS1	G1OpS2, G1OpS3, G2OpS2	G2OpS1						G1OpS2, G1OpS3
**G3OpS2**	G2OpS2	G2OpS2, G3OpS3	G1OpS2, G1OpS3, G2OpS2, G2OpS3	G1OpS2, G2OpS2	G1OpS2		G1OpS1, G2OpS3	G2OpS1		
**G3OpS3**		G2OpS2, G3OpS2	G1OpS3, G2OpS3	G1OpS2, G1OpS3, G2OpS3	G1OpS3		G1OpS1			
